# Children born during the hunger season are at a higher risk of severe acute malnutrition: Findings from a Guinea Sahelian ecological zone in Northern Ghana

**DOI:** 10.1111/mcn.13313

**Published:** 2022-01-10

**Authors:** Engelbert A. Nonterah, Paul Welaga, Samuel T. Chatio, Sarah H. Kehoe, Winfred Ofosu, Kate A. Ward, Keith M. Godfrey, Abraham R. Oduro, Marie‐Louise Newell, as members of the INPreP study group

**Affiliations:** ^1^ Navrongo Health Research Centre Ghana Health Service Navrongo Ghana; ^2^ Julius Global Health, Julius Center for Health Sciences and Primary Care, University Medical Center Utrecht Utrecht University Utrecht The Netherlands; ^3^ School of Medicine and Dentistry C K Tedam University of Technology and Applied Sciences Navrongo Upper East Region Ghana; ^4^ MRC Lifecourse Epidemiology Unit and NIHR Southampton Biomedical Research Centre, University Hospital Southampton NHS Foundation Trust University of Southampton Southampton UK; ^5^ Upper East Regional Health Directorate, PMB Bolgatanga Ghana; ^6^ Global Health Research Institute, School of Health and Human Development University of Southampton Southampton UK; ^7^ Department of Human Development University of Southampton Southampton UK; ^8^ School of Public Health, Faculty of Health Sciences University of the Witwatersrand Johannesburg South Africa

**Keywords:** food insecurity, Ghana, malnutrition, severe acute malnutrition, seasonal variation

## Abstract

Heightened food insecurity in the hunger season increases the risk of severe acute malnutrition (SAM) in childhood. This study examined the association of season of birth with SAM in a Guinean Sahelian ecological zone. We analyzed routine health and sociodemographic surveillance data from the Navrongo Health and Socio‐demographic Surveillance System collected between 2011 and 2018. January–June, the period of highest food insecurity, was defined as the hunger season. We defined moderate acute malnutrition as child mid‐upper arm circumference (MUAC) between 115 mm and 135 mm and SAM as MAUC ≤ 115 mm. We used adjusted logistic regression to quantify the association between the season of birth and SAM in children aged 6–35 months. From the 29,452 children studied, 24% had moderate acute malnutrition. Overall, 1.4% had SAM, with a higher prevalence (1.8%) in the hunger season of birth. Compared with those born October–December, adjusted odds ratios (aOR) and 95% confidence interval (95% CI) for SAM were increased for children born in the hunger season: January–March (1.77 [1.31–2.39]) and April–June (1.92 [1.44–2.56]). Low birth weight, age at an assessment of nutritional status, and ethno‐linguistic group were also significantly associated with SAM in adjusted analyses. Our study established that being born in the hunger season is associated with a higher risk of severe acute malnutrition. The result implies improvement in the food supply to pregnant and lactating mothers through sustainable agriculture or food system change targeting the hunger season may reduce the burden of severe acute malnutrition.

## Key Points

- Children born in the hunger season are at a higher risk of severe acute malnutrition in this Guinea‐Sahelian ecological zone.
- Children born with low birth weight were at a higher risk of severe acute malnutrition Being female and of the Nankam ethno‐linguistic group increased ones odds of severe acute malnutrition.
- Our observations imply sustainable food supply to pregnant and lactating mothers through improved agriculture or food system change targeting the hunger season may reduce the burden of severe acute malnutrition.

## Graphical abstract

Children born in the hunger season are at a higher risk of severe acute malnutrition. Low birth weight, female and Nankana children were likely to present with higher severe acute malnutrition. Improved food availability in the hunger season will reduce the burden of severe acute malnutrition.

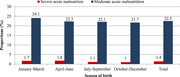

## 1 INTRODUCTION

After decades of undernutrition, Africa is now seeing a surge in overnutrition (Bain et al., 2013; Davies et al., 2018; UNICEF World Health Organization & World Bank, 2020). Despite the surge in overnutrition and the double burden, undernutrition remains an important public health problem in many African countries and requires a multidisciplinary approach to address it (Doku & Neupane, 2015). Like many countries in sub‐Saharan Africa (SSA), Ghana has a high burden of malnutrition among children aged 0–59 months with stunting, underweight and wasting all being highly prevalent (19%, 11%, and 5%, respectively in 2014) (Black et al., 2008; Ghana Statistical Service Ghana Health Service & ICF International, 2014), with highest rates in the northern part of Ghana (Ghana Statistical Service Ghana Health Service & ICF International, 2014; Yawson et al., 2017). In sub‐Saharan African countries most communities rely on rain‐enabled agriculture due to minimal access to irrigation facilities (Abizari et al., 2017; Chikhungu & Madise, 2014; Fentahun et al., 2018; Hagos et al., 2014). This creates a seasonal pattern of access to food, where the “hunger season” exposes families to food insecurity and its profound effect on nutritional status, particularly in children under age 5 years (Belayneh et al., 2020; Fentahun et al., 2018). The hunger season, a period between planting and harvest when food runs out is usually the worst hit period. This comes with profound adverse health effects on mothers and children. The hunger season is a challenging period for subsistence farmers and their families, who solely rely on what they grow.

The established underlying causes of malnutrition across all age groups likely follow a seasonal pattern, with consequences for morbidity and mortality across the life cycle (Bryson et al., 2021). Hence establishing the seasonal patterns in food availability and its effect on nutritional health or morbidity have policy implications in planning the distribution of scarce resources. While Demographic and Health Surveys do not measure the impact of seasonal variations in food supply and nutritional status, several epidemiological studies (though not at the country level) have provided insights (Sorgho et al., 2016). Most of these studies were not, however, conducted during the first 1000daysPlus (preconception through pregnancy, delivery and post‐delivery and infancy up to 5 years), which is considered by the World Health Organization as a critical period for optimizing growth. Further evidence accruing from the developmental origins of health and diseases concept further highlights the importance of the 1000daysPlus period as a highly vulnerable period where adverse exposures such as malnutrition can have lasting effects on health in later life (Davies et al., 2018).

Previous studies from SSA have reported inconsistent findings regarding any link between seasonal fluctuations in access to food and the nutritional status of young children (Chikhungu & Madise, 2014). Some studies from Gambia (Tomkins et al., 1986) and Malawi (Chikhungu & Madise, 2014) have reported seasonal trends in child growth and demonstrated weight and height deficits during the rainy season (period for planting with minimal food availability). A study from northern Ghana had also observed a seasonal effect of low dietary diversity and hence low micronutrient intake during the hunger season for both mothers and children (Abizari et al., 2017). In contrast, a study in Kenya observed increased nutrient intake in the rainy season. The preharvest season is often seen as a vulnerable period for acute malnutrition among exposed children. The effect is especially felt in drought‐prone areas which rely mostly on rain‐enabled agriculture or subsistence farming.

These current analyses of children aged 6–35 months from the Navrongo Health and Demographic Surveillance System (HDSS) (Oduro et al., 2012) was conducted as part of a broader INPreP ‐ Improved nutrition preconception, during pregnancy and post‐delivery study (https://www.southampton.ac.uk/global-health/research/lifecourse-epidemiology/inprep/about-us.page). We set out to determine the seasonal variation in the burden of severe acute malnutrition. We further determined the association of season of birth with SAM focusing especially on the effect of being born during the hunger season and the risk of SAM. We also reported on the unique factors in the Guinean Sahelian ecological zone and established factors that influence the nutritional status of children (Bain et al., 2013; Madan et al., 2018).

## 2 METHODS

We used data from the Navrongo Health and Demographic Surveillance system (HDSS) in Ghana (Oduro et al., 2012) for this study. As part of data collection in the HDSS catchment area, trained field workers visited all households 2–3 times a year to collect relevant data. The HDSS currently monitors 165,000 individuals; data collected includes pregnancies, births, deaths (through verbal autopsies), hospitalization, immunization, and migration. In addition, the field workers assess the nutritional status of children between 6 and 35 months by measuring their mid‐upper arm circumferences (MUAC). Other data collected include sociodemographic characteristics of the mothers and children including sex, age, maternal education, and household socioeconomic status (in wealth quintiles). Data on birth weight were extracted from the child health records of the children or the maternal health record book. For this study, we extracted and utilized HDSS data from 2011 to 2018.

### 2.1 Study setting

The Navrongo Health and Demographic Surveillance System (HDSS) (Oduro et al., 2012) operational area lie in an ecological belt that is food insecure due to climatic conditions. The area is highly dependent on rain‐enabled seasonal subsistence agriculture for livelihoods. We conducted this study in the Kasena–Nankana West district and Kasena–Nankana municipality of Northern Ghana. The study area lies within the Guinea‐Sahelian ecological zone in Northern Ghana, which shares a border with Burkina Faso in the north‐eastern corner. It covers a land area of 1675 km^2^ with an estimated population of 165,000 in over 32,000 households under continuous demographic surveillance. There are two main ethnic groups, the Kasena and Nankani, who together constitute about 96% of the population.

The main occupation of the people is rain‐enabled subsistence agriculture, and many parts (about 80%) are rural (Oduro et al., 2012). The study area faces cyclic food insecurity that alternates between pre‐ and postharvest seasons, making the population vulnerable to seasonal food shortages. The single rainy season in rural Northern Ghana often begins in May and ends around October. The area usually experiences heavy rainfall in August and September. Before some health interventions were introduced in the study area by the Navrongo Health Research Center (NHRC) both childhood and adult mortality were among the highest in the country. Under‐five mortality rates, as well as family sizes, have since declined considerably in the study area. The NHRC manages the Navrongo HDSS which monitors morbidity and mortality of the entire population in the study area. The HDSS collects and updates data on births, deaths, pregnancies, marriages, in‐migrations, out‐migrations, and other vital events by visiting households 2–3 times annually (Oduro et al., 2012). Through key informants in the community, new houses are identified for which field workers visit and then determine and register the number of households as well as resident individuals. These are then subsequently visited every 6 months to update the records and collect new information when needed.

### 2.2 Data management and statistical analyses

Routine data collected by the field workers are doubly entered and verified by a data manager for inconsistent entries using Visual FoxPro. We extracted the verified data and used STATA 16.0 for data cleaning and analysis. This analysis was limited to children with at least one MUAC measurement between January 2011 and December 2018. During each household visit, the mid‐upper arm circumference (MUAC) of each child below 3 years was measured. We used the first MUAC score of the child if the child had more than one. MUAC of <115 mm was categorized as severe acute malnutrition (SAM), MUAC between 115 mm and 135 mm as moderate acute malnutrition, and a MUAC of >135 mm as well‐nourished (World Health Organization and UNICEF, 2009).

Birth weight was categorized as low (birth weight <2.5 kg) or normal (birth weight ≥2.5 kg) and according to 11th edition of the World Health organizations' (WHO) international classification of diseases (WHO, 2019). Season of birth was grouped into four categories to reflect the weather patterns and food availability within a year, that is, January–March (postharvest season), April–June (immediate pre‐planting), July–September (planting season), and October–December (immediate harvest season). The hunger season was thus defined as the period between January and June, corresponding to the period of severely reduced availability of food.

We computed household wealth index using principal component analysis (PCA) from 30 separate household items, from large assets (e.g., land and car ownership) to smaller household items (e.g., phone, fan ownership). The principal component analysis of the household assets or items was done to predict factor scores. These scores were then categorized into wealth quintiles: Q1 = poorest, Q2 = poorer, Q3 = poor, Q4 = less poor, and Q5 = least poor.

We used logistic regression to examine the association between seasonality and severe acute malnutrition. Unadjusted and adjusted odds ratios (aOR) with 95% confidence intervals (95% CIs) were computed. We included all potential confounding variables in our data in our multivariable logistic regression model. These variables include season of birth, sex of child, birth weight, maternal education, wealth quintile, age of child, maternal age, mother's marital status, place of residence, and ethnicity. We did not find any statistically significant interaction between variables. The test of statistical significance was set at 5% (*p* < 0.05).

## 3 RESULTS

A total of 29,452 children aged 6–35 months and born between 2008 and 2018 were included in the analysis. Their median age was 11 months (interquartile range: 8–18). About 9% of the children had low birth weight, 50.2% were male and over half (55%) were age <12 months. The majority of the children (68.2%) were born to mothers aged 20–34 years, and 87% were residents in rural parts of the two districts. Almost 23% of the children were born to mothers with no formal education and 17.6% were born to mothers with secondary or tertiary education. Children born to unmarried women constituted 4% of the study population (Table 1)

**Table 1:** Background characteristics by severe acute malnutrition rate of children age 6–35 months included in the analysis in the Navrongo Health and Demographic Surveillance System from 2011 to 2018

Variable	Number (%)	Number with SAM	Percent with SAM	*p*‐value
Month of birth				
January–March	6479 (22)	107	1.7	**<0.001**
April–June	7871 (26.7)	141	1.8	
July–September	7656 (26)	88	1.1	
October–December	7446 (25.3)	72	1.0	
Sex				
Male	14,778 (50.2)	182	1.2	**0.023**
Female	14,674 (49.8)	226	1.5	
Birth weight				
Low birth weight	2615 (8.9)	62	2.4	**<0.001**
Normal birth weight	20,402 (69.3)	271	1.3	
Don't know	6435 (21.8)	75	1.2	
Maternal education				
No education	6683 (22.7)	93	1.4	0.82
Primary‐JSS	17,591 (59.7)	248	1.4	
Secondary‐tertiary	5178 (17.6)	67	1.3	
Household wealth				
Poorest	8345 (28.3)	106	1.3	**0.011**
Poorer	6802 (23.1)	109	1.6	
Poor	5500 (18.7)	94	1.7	
Less poor	5584 (19)	69	1.2	
Least poor	3221 (10.9)	30	0.9	
Age at measurement				
Under 1 year	16,240 (55.1)	287	1.8	**<0.001**
1–2 years	8846 (30.0)	102	1.2	
2–3 years	4366 (14.8)	19	0.4	
Maternal age				
<20 years	3291 (11.2)	35	1.1	0.108
20–34	20,075 (68.2)	276	1.4	
35–49	6086 (20.7)	97	1.6	
Marital status				
Married	28,317 (96.1)	388	1.4	0.268
Not married	1135 (3.9)	20	1.8	
Residence				
Urban	3740 (12.7)	44	1.2	0.242
Rural	25,711 (87.3)	364	1.4	
Ethno‐linguistic group				
Kasem	14,427 (49)	170	1.2	**0.006**
Nankam	13,686 (46.5)	221	1.6	
Buli	608 (2.1)	11	1.8	
Other	731 (2.5)	6	0.8	
Total	**29,452**	**408**	**1.4**	

*Note*: *p*‐value calculated using Pearson's *χ* ^2^ test to show differences between the groups of the variables in the row data.

Abbreviations: JSS, junior high school; SAM, severe acute malnutrition.

**Figure 1:**
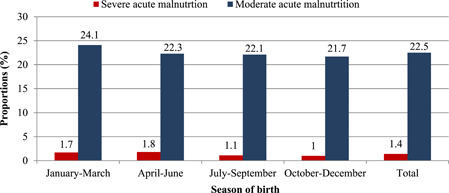
Proportion of children aged 6–35 months with severe acute malnutrition according to seasons in the Navrongo Health and Demographic Surveillance Site from 2011 to 2018

Figure 1 shows the malnutrition rates by season of birth. Overall 24% of children were malnourished, with 1.4% having SAM and 22.5% moderate acute malnutrition; 76% were well‐nourished. Children born in the second half of the hunger season, that is, April to June, had the highest rate of SAM (1.8%), while those born in the season after the harvest period (October–December) had the lowest SAM rate (1.0%). Low birth weight children had a SAM rate of 2.4% compared with 1.3% for normal birth weight children. Additionally, the SAM rate was 1.5% among females and 1.2% among males. Children aged below 12 months had a SAM rate of 1.8%.

**Figure 2:**
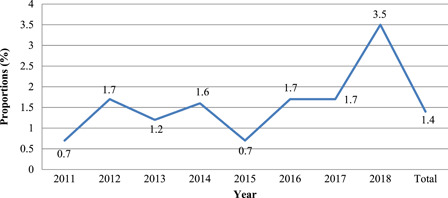
Yearly proportions of severe acute malnutrition cases in children aged 6–35 months in the Navrongo Health and Demographic Surveillance Site from 2011 to 2018

Figure 2 shows the yearly proportion of SAM cases among children 6–35 months in the NHDSS from 2011 to 2018. 2011 and 2015 recorded the lowest proportions of deaths while 2018 had the highest proportion. Mortality was fairly similar for the other years.

In Table 2, we show crude (OR) and aOR of sociodemographic characteristics, season of birth, economic characteristics of the mother and child characteristics in relation to SAM. In crude analyses, being born between January and June (hunger season), a female child, low birth weight, less than 2 years of age at the time of the survey, and coming from the Nankam ethno‐linguistic group all increased odds of SAM. In adjusted multivariable analyses, being born between April and June was associated with nearly two‐fold increased odds for SAM (aOR = 1.92, 95% CI [1.44–2.56]) compared to being born in October–December. Similarly, being born in the second half of the hunger season, January–April was associated with 1.8‐fold increased odds of SAM compared with being born in October–December (aOR = 1.77, 95% CI [1.31–2.39]).

**Table 2:** Crude and adjusted odds ratios of factors associated with severe acute malnutrition in children aged 6–35 moths in the Navrongo Health and Demographic Surveillance System from 2011 to 2018

Variable	Number (%)	Crude OR (95% CI)	*p*‐value	Adjusted OR (95% CI)	*p*‐value
Month of birth					
January–March	6479 (22)	1.72 (1.27–2.32)	**<0.001**	1.77 (1.31–2.39)	**<0.001**
April–June	7871 (26.7)	1.87 (1.40–2.49)		1.92 (1.44–2.56)	
July–September	7656 (26)	1.19 (0.87–1.63)		1.19 (0.87–1.63)	
October–December	7446 (25.3)	Ref		Ref	
Sex					
Male	14,778 (50.2)	Ref	**0.024**	Ref	**0.041**
Female	14,674 (49.8)	1.25 (1.03–1.53)		1.23 (1.01–1.50)	
Birth Weight					
Low birth weight	2615 (8.9)	1.80 (1.36–2.38)	**<0.001**	1.88 (1.42–2.50)	**<0.001**
Normal birth weight	20,402 (69.3)	Ref		Ref	
Don't know	6435 (21.8)	0.88 (0.68–1.13)		1.06 (0.81–1.38)	
Maternal education					
No education	6683 (22.7)	Ref	0.821	Ref	0.636
Primary/JSS	17,591 (59.7)	1.01 (0.8–1.29)		1.10 (0.86–1.42)	
Secondary/Tertiary	5178 (17.6)	0.93 (0.68–1.27)		1.18 (0.83–1.67)	
Wealth quintile					
Poorest	8345 (28.3)	Ref	**0.012**	Ref	**0.035**
Poorer	6802 (23.1)	1.27 (0.97–1.66)		1.25 (0.96–1.64)	
Poor	5500 (18.7)	1.35 (1.02–1.79)		1.36 (1.03–1.81)	
Less poor	5584 (19)	0.97 (0.72–1.32)		0.99 (0.72–1.36)	
Least poor	3221 (10.9)	0.73 (0.49–1.10)		0.73 (0.44–1.20)	
Age at measurement					
Under 1 year	16,240 (55.1)	4.12 (2.58–6.56)	**<0.001**	4.05 (2.52–6.51)	**<0.001**
1–2 years	8846 (30.0)	2.67 (1.63–4.36)		2.64 (1.61–4.33)	
2–3 years	4366 (14.8)	Ref		Ref	
Maternal age					
<20 years	3291 (11.2)	Ref	0.11	Ref	0.068
20–34	20,075 (68.2)	1.30 (0.91–1.85)		1.40 (0.98–2.00)	
35–49	6086 (20.7)	1.51 (1.02–2.22)		1.62 (1.08–2.43)	
Marital status					
Married	28,317 (96.1)	Ref	0.269	Ref	0.449
Not married	1135 (3.9)	1.29 (0.82–2.03)		1.20 (0.75–1.90)	
Residence					
Urban	3740 (12.7)	Ref	0.243	Ref	0.270
Rural	25,711 (87.3)	1.21 (0.88–1.65)		0.81 (0.55–1.18)	
Ethno‐linguistic group					
Kasem	14,427 (49)	Ref	**0.007**	Ref	**0.025**
Nankam	13,686 (46.5)	1.38 (1.13–1.68)		1.36 (1.10–1.69)	
Buli	608 (2.1)	1.55 (0.84–2.86)		1.55 (0.83–2.88)	
Other	731 (2.5)	0.69 (0.31–1.57)		0.84 (0.36–1.96)	

Abbreviations: CI, confidence intervals; JSS, junior high school and OR, odds ratios.

Children with low birth weight had an independent 90% increased odds of SAM compared to those with normal birth weight, (aOR = 1.88, 95% CI [1.42–2.50]). Children aged below 12 months at the time of the survey had four times greater odds of SAM compared to those aged 2 years or more, aOR = 4.05, 95% CI [(2.52–6.51]. Being aged 12‐23 months at the time of survey was associated greater than 160% increased odds for SAM compared to those aged two years or more (aOR = 2.64, 95% CI [1.61–4.33]). Females had 1.2‐fold increased odds for SAM compared to males (aOR = 1.23, 95% CI [1.01–1.50]). Children from the Nankani ethno‐linguistic group had a higher odds of SAM compared with the Kasem ethno‐linguistic group (Table 2).

## 4 DISCUSSION

We showed an overall SAM rate of 1.4% and a moderate acute malnutrition rate of 22.5%. The highest SAM rate by a season of birth was observed in children born between April and June, the period considered to be the severe hunger season. This is the immediate pre‐planting period and several months postharvest season. During this period most farmers are usually actively cultivating their farms and preparing to plant their crops. The INPreP study, a global health research group, had previously reported in a qualitative study that communities in the Navrongo HDSS see food insecurity as a major challenge to optimal nutrition due to lack of irrigated agriculture and improved agriculture (Debpuur et al., 2020; Watson et al., 2020). The highest rate of SAM was observed in 2018 and this may be due to the worse climatic conditions leading to poor yield and harvest. Fagariba et al. (2018) in study conducted in a neighboring Talensi district observed that 2018 experienced highest draught and temperature hence affecting food production despite climate change adaptation practices such as a change in time of planting, improved crop varieties and land rotation. Low birth weight children were most likely to be severely malnourished compared children with birth weight greater than 2.5 kg.

In multivariable analysis, we found that being born between April and June, being female, having low birth weight (birth weight<2.5 kg), born into a poor household, being less than one year old and being from the Nankam ethnic group in the study area conferred the highest odds for SAM. The season in which a child is born is important to the child's growth and health. Agriculture serves as the primary livelihood of the people with a majority of the inhabitants being subsistence farmers who live in small scattered settlements. In northern Ghana, the agricultural cycle is driven by agro‐ecological conditions and by annual cycles in the weather. Our study showed that children born between April and June, which is considered to be the lean season, had increased odds for SAM compared to children born during the harvest period of July to September and the immediate postharvest period of October to December. It is possible that during the hunger season with food insecurity, the diet of a lactating mother may not be optimal and could therefore affect the child's adequate lactation which could acutely affect nutritional status of the child. Previous studies in sub‐Saharan Africa have also observed the association of seasonal variation with incidence of low birth weight and severe acute malnutrition (Rogawski McQuade et al., 2019).

Previous studies have shown that the mother's diet in pregnancy and lactation may program her offspring where the fetus adapts to undernutrition by changing its metabolism, altering its production of hormones and sensitivity of tissues to them, redistributing its blood flow, and slowing its growth rate (Morrison & Regnault, 2016). Children born in the harvest period of October–December had the lowest SAM rate, perhaps due to the good supply of food and hence higher nutritional status of the lactating mother.

Our results showed a strong association between birth weight and SAM among children in the population. The odds ratios of SAM in low birth weight children was nearly twice the odds of severe acute malnutrition in normal birth weight children. This observation is consistent with similar studies conducted in Tanzania (Rogawski McQuade et al., 2019), Malawi (Chikhungu & Madise, 2014), Ethiopia (Fentahun et al., 2018), India (Choudhary et al., 2019), and Bangladesh (Hillbruner & Egan, 2008). One of the major reasons for the association of low birth weight with SAM is suboptimal maternal nutrient intake. In the hunger season, there is greater likelihood that milk production among lactating mothers is reduced (Roba et al., 2016). Children born in the lean season also have a greater susceptibility to weight loss in the first two weeks of life and this may also contribute to the higher risk of SAM. This may be explained by low quantity and/or quality of breast milk during this time. Some studies observed that these disparities were persistent and associated with smaller but sustained deficits at the age of 2 years. This suggests incomplete catch‐up growth among children born in the high food insecurity season. Low birth weight children may also be prone to infection (such as diarrheal diseases and respiratory infections), loss of appetite and fatigue, jaundice, and anemia (Jones & Berkley, 2014). These competing morbidities may further hinder catch‐up growth among these children and set them on a trajectory of sustained malnutrition and poor physical growth till after the age of five years.

In the study area, the hunger season is also associated with harsh climatic conditions including the seasonal north‐east harmattan winds blowing from the Sahara and extremely high temperatures, which may affect dry season gardening due to lack of irrigated agriculture. These may act together to increase respiratory infections and further worsen nutrient intake in the said period. The Ghana Nutrition profile shows that younger children are at a higher risk of SAM but data on age‐categorized sub‐analysis was not provided (https://www.usaid.gov/sites/default/files/documents/1864/Ghana-Nutrition-Profile). Largely consistent with our findings, a study from northern Ghana had previously established that malnutrition was higher among children aged 6–8 and 12–23 months (Saaka et al., 2015). The authors observed that the lower age groups (<12 months) were less likely to recover from severe acute malnutrition (Akparibo et al., 2017).

We observed that lower household socioeconomic status was associated with higher risk of severe acute malnutrition while relatively wealthier households having lower risk of SAM. Similar findings have been previously reported in studies from western Nigeria (Imam et al., 2020), Pakistan (Ahmad et al., 2020), and Jamaica (Thompson et al., 2017). A study from Nepal also observed that families with higher socioeconomic status were likely to present with lower SAM (Hossain et al., 2020). These findings were further corroborated by a study in Ghana that observed that lower family income was associated with high levels of SAM (Tette et al., 2016). A plausible reason is that higher economic status is associated with a higher purchasing power and hence access to food during the lean season. This may thus maintain a good or healthy dietary diversity across the seasons.

We further observed that maternal education was not associated with SAM. This has previously being observed in a study from Bangladesh, where in addition to maternal education, variations in household income did not influence levels of SAM (Rahman et al., 2016). The importance of the influence of maternal education with malnutrition is important because education is known to influence maternal food choices, practices of good sanitary conditions and prevention of infection. Though access to formal education to the girl child is still a burning issue in some countries in SSA, our findings may result from the absence of indirect factors that mediate the relation of education to SAM.

We observed female children were likely to be severely malnourished during the hunger season. Consistent with our findings was findings from a study from Nepal that previously reported that male children were less likely to have SAM compared with female children (Hossain et al., 2020). This was further observed by a study in Tanzania (Rogawski McQuade et al., 2019) but contrary to findings from 16 demographic health surveys reports from SSA (Wamani et al., 2007). A further systematic review also reported boys are more vulnerable to malnutrition and stunting compared with girls (Thurstans et al., 2020). These inconsistent findings may suggest the varying reasons currently provided for these differences are more speculative rather than informed by direct evidence. Further longitudinal studies may be required to explain the observed findings.

Overall our findings have important implications for policies and program implementers in the study area. These results suggest that children, particularly girls, born in the hunger season are vulnerable to SAM and hence poor health outcomes. The implications may extend into adulthood as severe malnutrition and poor growth have been associated with reduced cognitive function and risk of chronic diseases later in life (Liu et al., 2003). Prenatal care interventions, including nutritional supplementation, could be targeted during high food insecurity months to reduce seasonal disparities. Implementing initiatives to promote food market development and improve economic prospects especially during the hunger season are likely to help prevent seasonal declines in nutritional status. Further studies are also needed to elucidate the possible reasons for sex differences in nutritional status.

### 4.1 Strengths and limitation

Our study does have several strengths including a large representative sample size which makes our results applicable to the HDSS study area. Our study also highlights the need to tailor interventions to the most vulnerable periods in the year. We would like to highlight a few limitations of our study. Assessing the effect of seasonal variability severe acute malnutrition poses methodological challenges. These include exposure assessment, ecological fallacies and the complexity of relationships. Ecological fallacy in our study may be minimized by the minimal local variations in seasons. However, residual confounding may still persist due to some unmeasured factors that may influence the findings of this study, and hence interpretations should be done with caution.

The use of crude MUAC measurements unadjusted for age may lead to misclassification of some participants and may also affect the rates of malnutrition reported. The World Health Organization growth and development classifications (Z‐scores/norms) are usually criticized for their inappropriateness in some SSA contexts due to potential misclassifications. There are currently conflicting reports with several studies reporting agreement between waist‐for‐height (WHZ) scores and MUAC while other studies have reported disparities (Mehta et al., 2013). This implies the scores perform differently in different populations. However, MUAC measurements have been validated and found to be viable, low‐cost, low‐burden alternative for community‐level nutritional status assessment.

## 5 CONCLUSION

We established that being born in the hunger season is associated with a higher risk of SAM in this Guinea‐Sahelian ecological zone. The result implies improvement in the food supply to pregnant and lactating mothers through sustainable agriculture or food system change targeting the hunger may reduce the burden of SAM. Further work is also needed to understand the mechanisms underlying these observed associations, especially the role of the pregnancy/lactation period so as to guide the development of appropriate interventions.

## CONFLICT OF INTERESTS

The authors declare that there are no conflict of interests.

## ETHICS STATEMENT

Ethical approval for the operations of the Navrongo HDSS was obtained from the Navrongo Health Research Centre's Institutional Review Board (NHRC‐IRB). An additional ethics approval for the analyses of the data through the INPreP project was obtained from the NHRC‐IRB (approval number NHRCIRB325).

## AUTHOR CONTRIBUTIONS

EAN, KMG, and M‐LN designed the INPreP study. EAN and PW conceived the paper. PW analyzed the data and EAN interpreted the results and wrote the first draft. PW, STC, SHK, KAW, KMG, and M‐LN contributed to writing the paper by commenting on successive drafts. ARO is head of the Navrongo Health and Socio‐Demographic Surveillance System and WO is head of Upper East Regional Health Directorate and both are responsible for the data. All authors critical reviewed the paper and approved the final draft for submission.

## Data Availability

Data used in this paper are available upon reasonable request from the corresponding author. The Navrongo Health and Demographic Surveillance System data can be obtained through a request to the Navrongo Health Research Centre's director (https://www.navrongo-hrc.org).
